# Reproductive Health Needs Assessment in the View of Iranian Elderly Women and Elderly Men 

**Published:** 2018-03

**Authors:** Mahsa Shakour, Kobra Salehi, Nikoo Yamany

**Affiliations:** 1 Medical Education Development Centre, Arak University of Medical Sciences, Arak, Iran; 2 Department of Nursing and Midwifery, Isfahan University of Medical Sciences, Isfahan, Iran; 3 Medical Education Research Center, Isfahan University of Medical Sciences, Isfahan, Iran

**Keywords:** Geriatric, Sexual Health, Needs Assessment

## Abstract

**Objective:** To assess reproductive health needs in men and women as the first and basic step in educational planning.

**Materials and methods:** The study was qualitative. Participants were Postmenopausal women and men over 60 years. Data gathering was done by semi structured interviews. Analysis of the qualitative data was conducted during a multi-step process according to the assessment method of Altschuld et al needs analysis.

**Results:** Two general themes were considered in categorizing codes extracted from interviews: 1) Problems, 2) Demands. Women’s reproductive health problems were Problems associated with menopause, Related to family planning, sexual problems, and diseases and cancers. Reproductive health problems in men were in two main subthemes Urinary-reproductive problems and sexual dysfunction. Their main demand was for establishing a health center for geriatric reproductive health.

**Conclusion:** Aging has severe effect on men’s and women’s reproductive health and elderly peoples need health services to cope with changes, therefore these needs should be considered in medical curriculums.

## Introduction

Increasing life expectancy along with decreased birth rate and population growth rate in recent years have caused increased elderly population over recent decades. WHO in a message entitled "Elderly People as a New Force in the Way of Development" published to mark International Day for the Elderly in 2003 noted that today a revolution is to happen in demography at global level. Although aging is a continuous process in human life, it is not clear at which age it exactly starts, and there is no single definition regarding its initiation time (1). However, most urban societies consider border age as 60, i.e. when the person is retired, as start of elderly, and people above 60 are recognized as elderly of the society (2, 3). Current growth in the elderly population is annually 2.4%, which is clearly more than the overall growth rate of the population. Currently about 600 million people are 60 years and older in the world and this figure doubles by 2025, that is, it reaches to 1,200 million people (1, 4). According to the latest census in 2011, 6,165,676 elderly people over 60 years live in the country, which constitutes 8.2% of the country's total population (5).

Given the fact that aging population will impact on health and social policies, the elderly people will be among the policy making priorities in the few next decades (6). This fact should always be taken into account in national health programs that increased life expectancy should be accompanied by the health and it should not merely lead to increased lifelong along with sickness and disability. Attention to preserving and promoting the health as well as treatment and control of some disease is especially important in this age group.2Considering the problems and the aging of population, further studies about the health care should be done in this age group (4).

The issue is more complicated regarding women and especially their reproductive health. In addition to common aging problems, women experience Menopause before aging. Awareness and knowledge of women and family members as well as of the society should be increased for adaptability and understanding changes resulting from Menopause. The average age of menopause onset is 51 years (7). Considering increased life expectancy in the world and Iran which was reported as 72.1 years for women, women spent about one third of their life in Postmenopausal period (5, 8). This time is roughly equivalent to the time when risk of chronic diseases and Breast cancer are increased considerably (7, 9, 10) Researchers believe menopause is a complex phenomenon which requires simultaneous biological, psychological and social changes in lives of women. On the other hand, menopause meaning and its perception by women considerably influence self-care and follow-ups in this period. One of the problems faced by health care providers is that there is no balance between understanding of women about menopause as a natural event and health care for this period which can prevent from chronic consequences and diseases. Perceptions, attitudes and knowledge about menopause, may vary in women in different populations. Despite of extensive studies on symptoms and signs of menopause, rare information is available on personal meanings or attitudes of women about menopause as experienced by Iranian women. Significance of this type of studies is proper description and interpretation of an experience which causes increased insight and leads to more and deeper thoughts on the subject (11, 12). Thus, investigating menopausal women in terms of these consequences in improved life quality in elderly and society health is crucially important. Considering increased number of menopausal women population and increased life expectancy and increased lifelong, identifying health problems and needs of these women is important, and caring for this increasing generation should be regarded as a necessity (13, 14). But few studies have addressed awareness of women regarding issues related to reproductive health especially their needs (9). Increasing the age and aging influences also men’s reproductive health. Traditionally men consider themselves as healthy people which suffer less health-related problems and it causes they seek for health aids less (15). Improved planning for men requires having accurate information concerning disorders related to the reproductive health of men. But there is no comprehensive information on the middle-aged and elderly men in Iran.

Concerns on health and its related behaviors should be studied in men and women so that information regarding reproductive health needs of these people is provided for health policy makers, service providers and education sector. However, health team has rare information on concerns and needs of elderly men and women (16) and a few studies have been conducted on reproductive health needs of elderly and aged women (6). On the other hand, in order to be successful in providing services to the elderly people, necessary competencies should be developed in health service providers. Thus, elderly people needs should be considered in educational planning for these people, and the first step is needs assessment. Since educational needs are the most important determinants in formulating and designing educational courses, educational content and generally health and education process at all levels of educational system (17). Needs assessment is done from various sources such as experts and service receivers. Regarding reproductive health needs assessment, expert ideas in other studies have been used (18, 19). In the current study, which aims at investigating needs related to reproductive health in men and women passing reproductive period, needs assessment was done from the society as the first and basic step in educational planning.

## Materials and methods

The study is a needs assessment which was conducted qualitatively. Postmenopausal women aged 45-65 years and men over 60 years living in Isfahan formed the study sample. Method of sampling was purposive and convenience. Sampling was continued until saturation was achieved. After getting permission from Isfahan University of Medical Sciences (the number of ethical approval is 389284) and selected two health centers (Shahid Aval and Navabsafavi), in coordination with the responsible centers of health, the health volunteers were asked to cooperate to facilitate the work. Using information available in health files and approval of volunteers, qualified women were selected and invited for interview in the certain day.

For higher efficiency, women were interviewed in two focus groups with six-members regarding reproductive health needs and problems. The women were after menopause that they were not of reproductive age (45- 65 years old). They were questioned in semi-structured manner and they were encouraged to express their reproductive health needs and problems and experiences for each other and state their ideas clearly. Thus, access to their knowledge and attitude was facilitated. Following gaining consent of people, all interviews were recorded and note taking was done and they were transcribed after the meetings (20).

 For men, information was collected using personal and semi-structured interview by male researcher. 10 men above 60 years old living in Isfahan who referred to selected health centers for screening and visited general practitioner or referred with their wives were interviewed. It took approximately 9 hours. Totally 7 persons had diploma, 2 persons were little-educated and one person didn’t have any literacy. 

For semi structured interviews, interviewer asked these questions: What are your experiences about sexual health? What are your problems for family planning? What are your problems about rules for reproductive health? What were your problems related to urinary and sexual systems, when you were 60 years old (for women 45 years old)? What are your requests? If you had access to an expert in reproductive health, do you refer him/her? Why?

Data were analyzed using a multistep process in accordance with the method used by Altshold et al. (21, 22). In the first step, only responses were considered without any data analysis, and they were shortened in a meaningful manner. In the second step, themes were extracted. Themes were initially categorized in different health areas as health problems or needs and preferences, and Identified data categories (IDCs) were developed. Cases extracted from previous steps were investigated with emphasis on IDC, and relevance to themes for each item was identified and themes were finalized with assuring correct and accurate analysis process. Thus, information obtained from interviews was extracted as qualitative variables and themes.

*Lincoln* and *Guba's* Evaluative *Criteria* were used for rigor, reliability and validity of data (23). That is, in order to make research acceptable, in addition to long term involvement of authors in data collection and analysis, review of research colleagues familiar with qualitative research and subject of research were also used.

## Results

Two general themes were considered in categorizing codes extracted from interviews: 1. Problems, 2. Demands. Reproductive health problems of men and women were put in different themes. Women’s reproductive health problems were categorized in following subthemes: 1) Problems associated with menopause, 2) Related to family planning 3) sexual problems, 4) diseases and cancers common in women (Table 1). 

Signs and symptoms of menopause, without relaxation after menopause, anxiety and palpitations were problems mentioned by postmenopausal women in sub-theme related to menopause. Expensive drugs on the market for treatment of the signs and symptoms associated with menopause, failure to identify a suitable treatment method for the treatment or prevention of complications, pre-specified prescriptions of physicians regardless of the patient were other issues and concerns of postmenopausal women.

A theme was “Problems associated with menopause” for this theme an old woman said: when I feel symptoms of menopause I feel very ill, I have heartthrob and I think I would be died…”

Other old woman said: “I feel these hormonal changes in my body, I mean that I feel burning…”

For subtheme “Failure to identify a suitable treatment method for the treatment” a woman said “every time they say a thing, one time they say you should take Omega3 and next time they say it is not useful!...” or other woman said: “I am not better after taking my medication”.

In sub-theme of sexual problems, such codes as lack of sexual pleasure, decreased libido due to chronic illness of oneself or the spouse and fear of chronic disease exacerbation, decreased libido followed by hysterectomy were extracted as needs in the interviews. Women stated financial problems are one of cases which lead to their psychological and mental problems and thus their libido is decreased.

In sub-theme of women’s common diseases and cancers, interviewed women stated they are not aware of the common causes and symptoms of cancers common in menopause period. 

**Table 1:** The problems of women’s reproductive health

**Subthemes**	**Codes**
Problems associated with menopause	Signs and symptoms of menopause Severity of signs of menopause like death Without relaxation after menopause Anxiety Palpitations Expensive drugs on the market for treatment of the signs and symptoms associated with menopause Failure to identify a suitable treatment method for the treatment Prevention of complications Pre-specified prescriptions of physicians regardless of the patient Forced hysterectomy to decrease the signs of menopause
Related to family planning	Unwanted pregnancy in menopause Fear of unintended pregnancy Side effects of tubectomy in elderly women Imagine the pain because of previous tubectomy False Pregnancy Diagnosis
Sexual problems	Lack of sexual pleasure Decreased libido due to chronic illness of oneself or the spouse Decreased libido due to fear of chronic disease exacerbation Decreased libido followed by hysterectomy Decreased libido followed by financial problems
Diseases and cancers common in women	Lack of knowledge of the common causes and symptoms of cancers common in menopause Lack of knowledge about pap smear during menopause and afterwards

For example, they had no information on Pap smear during menopause and afterwards.

Concerning stated problems, menopausal women expressed followings as their demands: providing such services as consulting on treatment of menopause signs and symptoms, medical insurance, self-relaxation education and better patient – doctor communication. Also, in relation with sexual issues, they expected some centers are introduced to them for consulting in this regards so that they utilize consulting services to decrease their sexual problems. They wanted to be familiar with prevention ways for cancers, their symptoms and signs and their diagnostic tests, and to be consulted regarding the cancers. 

Reproductive health problems in men were categorized in two main themes: 1) Urinary - reproductive problems and 2) sexual problems3) family planning (Table 2).

In sub-theme of urinary - reproductive problems, following codes were extracted from interviews with men: Urinary dysfunction, frequent urination, prostate enlargement, adverse effects of urinary dysfunction medications on sexual matters and neglecting screening test for prostate enlargement. In sub-theme of sexual problems, men stated they suffer from sexual dysfunction and their libido was decreased. Men believed medicines used for urethral stricture reduce Orgasm Dysfunction in them. Also, weight gain and obesity were mentioned as the causes of their orgasm dysfunction. Chronic diseases were also among cases mentioned by men that cause low libido in men. 

For example for subtheme “Adverse effects of urinary dysfunction medications on sexual matters”, an old man said: “when I was 55years old, urinary dysfunctions begun and these problems were less by medicines but I had problems for my sexual activities…” or other man said “when I took medicines I felt low libido…”.

A sub theme was “Fear of side effects of vasectomy”. Some men thought that some disease that they had was related to vasectomy. For example a Man for this subtheme said:” after vasectomy I had heart failure rate….” Or other man said “I feel I have low libido after vasectomy…”Similar to group of women, men stated financial problems for treatment of diseases and complications adversely influence their libido.

**Table 2:** The problems of men’s reproductive health

**Subthemes**	**Codes**
Urinary- reproductive problems.	Urinary dysfunction Frequent urination Prostate enlargement Adverse effects of urinary dysfunction medications on sexual matters Neglecting screening test for prostate enlargement
Sexual problems	Sexual dysfunction Decreasing libido Reducing orgasm dysfunction after taking medicines for urethral stricture Weight gain and obesity and orgasm dysfunction Chronic diseases and low libido. Vasectomy and low libido
Related to family planning	Refusing to use contraceptives Unintended pregnancy of their wives Fear of their wives' pregnancy Fear of side effects of vasectomy

## Discussion

The main aim of the study was investigating needs related to reproductive health in men and women passing reproductive period. Needs assessment was done from the society as the first and basic step in educational planning. Because of growing numbers of elderly need especial health care, we did this study. On the other hand, it is predicted elderly explosion will occur in Iran in 1410 and 25 – 30 percent of population will above 50 years old (24). Thus, elderly and aging of population needs increased studies on health and care needs of this group (4).

In fact, signs and symptoms of menopause are considered as most common complaints of women in menopause and postmenopausal period. These complications are frequently observed in most studies conducted on menopausal women (25).

Meanwhile, the most common complication is symptoms of vasomotor and flushing feet. Although complications of vasomotor do not threaten women’s life, it cause severe anxiety and discomfort in them and even adversely influences job of many of women (26, 27).

 In some cases, it causes lack of comfort in women (28). These symptoms may last up to 5 years and sometimes continues until 15 years after menopause (29-30). It is one of the problems which were mentioned by postmenopausal women under study. Despite menopause is a physiologic process which should put clients and health team beside each other and provide an opportunity for participation of women in their health trend and such participation may initiate a positive trend, not only such association was not observed in the study, but also according to their view, pre-written prescriptions of physicians regardless of the patient exacerbates the condition (31).

Presence of consulting centers for menopausal symptoms and its treatment were among needs mentioned by women. Despite other researchers also suggested establishment of such centers in their studies (32), authorities yet ignore this recommendation. In the study by Leon et al., less than 50 percent of women under study stated they didn’t have adequate information on menopause and majority of women wanted receiving information about menopause (12).

This need was also observed in findings by other studies. So that Sanagou and Juybari reported postmenopausal women in their study had received no consultation or training through medical and health programs (11). According to Landsburg, only a few percent of women in his study were aware of the effects of aging on reproductive health issues (9).

These information and training which include tests and screening common cancers in women in menopause period and postmenopausal period are important in early diagnosis of diseases, that women under study were not aware of them. Lack of awareness and familiarity with screenings is also observed in other communities (33).

Dyspareunia or painful intercourse due to atrophy of the urogenital system, along with dryness, irritation and burning during sex and afterwards are discussed causes of sexual dysfunction in the elderly (27,31) Sexual problems such as lack of orgasm, orgasm dysfunction and lack of guidance centers were problems mention by study women. In a review study during 1990 – 2008, sexual dissatisfaction and loss of libido were the most common sexual complaint among women (34). In fact many postmenopausal women suffer from sexual dysfunction, but its incidence and etiology is unknown (27). Libido is one of the main and health-related parts for feeling well in women at all ages. It has complex nature and is influenced by various factors (35). Most sexual experiences and sexual functioning in elderly women is highly dependent on basic conditions of woman such as overall health, physical and mental health and living situation (10, 25, 36).

In the Islamic perspective, Sex in the context of marriage is a legitimate, enjoyable activity and there is positive outlook toward it; but it is not a subject that is openly discussed. Cultural taboos dictate that sex should remain a private matter between husband and wife. This explains, at least in part, why Muslims are reluctant to seek help for sexual and the long time lag before seeing a physician (37).

Incompatibilities and conflicts with a spouse, insomnia, life stress and depression affect women's libido. In addition, sexual problems developed in men due to aging should not be ignored (38). Similar to the current study, women stated chronic diseases as a factor in reduction of their sexual functioning and libido in other studies (39).

Similarly, in a study on sexual dysfunction in the elderly, women reported that their physicians did not ask any questions about their sexual health, while they tended to speak about their needs with a physician (40). In fact, giving opportunity to women for discussion on sexual issues and problems should be main part of their health care and one should receive suitable information and attitude in support of sexual relationship in elderly ages (34, 41). However, client – physician relationship in sexual issues is yet poor. Reluctance to talk in this regard, negative attitude in the society regarding women’s sex in elderly may prevent from such discussions with the physician (41).

Weight gain and obesity were mentioned as the causes of men’s orgasm dysfunction. Urogenital problems, such as urinary dysfunction, frequent urination, prostate enlargement, side-effects of urinary dysfunction medications on sexual matters and neglecting screening test for prostate enlargement were other problems extracted from interviews with men. Hartmann and colleagues also pointed out that the incidence of sexual dysfunctions especially decreased libido increase by weight gain. High body mass index, abnormal lipid profiles in middle-aged men has been proposed as one of the effective factors of sexual dysfunction in the elderly (16).

Chronic diseases were mentioned as the causes of men’s orgasm dysfunction and low libido. In fact, some of diseases such as diabetes, hypertension and heart disease have known effects on male sexual dysfunction (42). Diseases and medications may affect sexual relations adversely and men in the study also pointed to it (16). By increasing the age, the body's homeostatic level of reserves is limited which leads to a reduction in clearance and increased toxics and side effects of many drugs and medications (40).

Similar to women, men stated financial problems for treatment and complications influence their libido. Studies which addressed behaviors related to elderly health services in developed countries, proposed income as an index for access to health related resources (6).

Neglecting screening test for prostate enlargement was one of the problems were extracted from interviews with men. Amongst men over 65 years, one out of nine men will suffer from Prostate Cancer (43). While this disease is predictable and controllable by men’s Prostate Cancer screening test. In the study by Holden et al., severe symptoms of lower urinary tract and prostate disease were reproductive health problems in men over 40 years and only half of them had done a screening test for prostate cancer and this makes clear the need to establish appropriate services and education strategies to improve reproductive health in these men. Although prostate diseases, urinary symptoms and erectile dysfunction are often ignored, they constitute main part of men’s reproductive health which occurs in elderly (44).

There are some studies about old women’s reproductive health needs in Iran, but they are limited. This study is the first needs assessment that investigates the elderly men’s reproductive health needs in Iran and could be used in medical curriculum planning. Old men and women shame to describe their personal experiences about problems in reproductive health, and this was the most prominent limitation in our study.

The researcher accepts limitations, such as the sample being drawn from one region, which may not be representative of other areas, the needs being vast for needs assessment, and also researcher couldn’t ask male samples, because of the type of issue and culture.

We make suggestions for future research needed in this field. We suggest to reform the current curriculum of medicine, midwifery and public health according to results of reproductive health could be useful for health promotion.

## Conclusion

Men and women’s reproductive health needs are important as much as other health needs. Not only the present study, but also other studies in other parts of the world indicate sometimes these needs may be totally ignored due to passing reproductive stage of women and men, while paying attention to them helps preserving health. 

To this end, findings of the current study suggest needs and problems related to reproductive health of postmenopausal women and elderly men and findings from more extensive studies may help health policy makers, service providers and education sector in promoting health services to these people.
